# Mcc1229, an Stx2a-Amplifying Microcin, Is Produced *In Vivo* and Requires CirA for Activity

**DOI:** 10.1128/iai.00587-21

**Published:** 2022-02-17

**Authors:** Erin M. Nawrocki, Laura E. Hutchins, Kathryn A. Eaton, Edward G. Dudley

**Affiliations:** a Department of Food Science, The Pennsylvania State University, University Park, Pennsylvania, USA; b Department of Microbiology and Immunology, University of Michigan, Ann Arbor, Michigan, USA; c *E. coli* Reference Center, The Pennsylvania State University, University Park, Pennsylvania, USA; University of Pennsylvania

**Keywords:** *Escherichia coli*, Shiga toxins, bacteriocins

## Abstract

Enterohemorrhagic Escherichia coli (EHEC) strains, including the foodborne pathogen E. coli O157:H7, are responsible for thousands of hospitalizations each year. Various environmental triggers can modulate pathogenicity in EHEC by inducing the expression of Shiga toxin (Stx), which is encoded on a lambdoid prophage and transcribed together with phage late genes. Cell-free supernatants of the sequence type 73 (ST73) E. coli strain 0.1229 are potent inducers of Stx2a production in EHEC, suggesting that 0.1229 secretes a factor that activates the SOS response and leads to phage lysis. We previously demonstrated that this factor, designated microcin 1229 (Mcc1229), was proteinaceous and plasmid-encoded. To further characterize Mcc1229 and support its classification as a microcin, we investigated its regulation, determined its receptor, and identified loci providing immunity. The production of Mcc1229 was increased upon iron limitation, as determined by an enzyme-linked immunosorbent assay (ELISA), *lacZ* fusions, and quantitative real-time PCR (qRT-PCR). Spontaneous Mcc1229-resistant mutants and targeted gene deletion revealed that CirA was the Mcc1229 receptor. TonB, which interacts with CirA in the periplasm, was also essential for Mcc1229 import. Subcloning of the Mcc1229 plasmid indicated that Mcc activity was neutralized by two open reading frames (ORFs), each predicted to encode a domain of unknown function (DUF)-containing protein. In a germfree mouse model of infection, colonization with 0.1229 suppressed subsequent colonization by EHEC. Although Mcc1229 was produced *in vivo*, it was dispensable for colonization suppression. The regulation, import, and immunity determinants identified here are consistent with features of other Mccs, suggesting that Mcc1229 should be included in this class of small molecules.

## INTRODUCTION

Enterohemorrhagic Escherichia coli (EHEC) strains are foodborne pathogens that can cause severe clinical complications, including hemorrhagic colitis (HC) and hemolytic-uremic syndrome (HUS), through the production of Shiga toxin (Stx) and other virulence factors (1–3). Stx is encoded on a temperate lambdoid bacteriophage and is therefore induced via the bacterial SOS response (4–6). Certain antibiotics and DNA-damaging agents are known to trigger phage induction and increase the expression of Stx *in vivo* and *in vitro* (7, 8). In the intestinal environment, members of the microbiome and their metabolites can modulate the pathogenicity of EHEC strains in multiple ways (9). Commensal bacteria can reduce the growth and colonization of EHEC, broadly limiting virulence factor expression (10). Alternatively, strains that are sensitive to the *stx*-converting phage can be infected and thus amplify Stx production (11–13). Finally, small molecules such as bacteriocins that target EHEC can both inhibit growth and promote Stx expression by the induction of the phage lytic cycle (14, 15).

Bacteriocin activity was first described nearly a century ago (16) and is widespread in E. coli, with up to 60% of strains being identified as colicin producers in some surveys (17–19). Microcins (Mccs), which have a lower molecular weight than colicins (20), are found less frequently and are not as well characterized (21). They are generally smaller than 10 kDa in size, are not SOS-induced, and are secreted by intact cells (22, 23). Foundational studies on microcin B17 (MccB17), MccJ25, and others revealed that microcins are typically expressed in stationary phase when cells are starved for nutrients (24–26). In particular, iron-limiting conditions often stimulate microcin production (27–29). Some microcins are posttranslationally modified with the addition of siderophores (30–32), and many colicins and microcins exploit siderophore receptors for entry into target cells (33, 34). The expression of bacteriocins in nutrient-poor environments can also confer a fitness advantage to producing strains, allowing them to kill their competitors and better colonize a given niche (35–37). In mouse models, for example, iron limitation can be advantageous for either pathogens (38) or probiotic bacteria (39) that produce bacteriocins.

Previous studies of the human E. coli isolate 0.1229 revealed that cell-free supernatants from this strain were sufficient to induce the SOS response and increase the Stx expression of EHEC (15). Microcin B17, which is encoded on a 96.3-kb plasmid in 0.1229, contributed to but was not fully responsible for SOS induction or Stx amplification (15). An additional factor with Stx-amplifying activity was localized to p0.1229_3, a 12.9-kb plasmid in the strain (15). This activity was dependent on TolC for efflux from 0.1229 and TonB for import into the target cell (15). The SOS-inducing, Stx-amplifying agent of p0.1229_3 is presumed to be a new microcin, first described in strain 0.1229 and thus designated Mcc1229. Although the chemical identity of Mcc1229 is not known, it is encoded within a 5.2-kb region of p0.1229_3 whose annotations include hypothetical proteins, an ABC transporter, a cupin superfamily protein, and domain of unknown function (DUF)-containing proteins (15).

Only a small number of microcins have been purified, and their functions in complex environments like the gut microbiome are not well defined (21). Some have theorized that the microcins prevalent in phylogroup B2 E. coli strains enhance their ability to dominate the rectal niche and colonize the urinary tract (40). 0.1229 is a phylogroup B2 isolate of sequence type 73 (ST73). Other members of ST73 are notable urinary pathogens (e.g., CFT073), and the lineage carries many virulence factors that can promote colonization and persistence *in vivo* (41, 42). In 0.1229, MccB17 and Mcc1229 may serve this purpose, as they are lethal to competing E. coli strains (15). To elucidate the role of the putative microcin Mcc1229, we have clarified its export, import, immunity, and regulation. We have also probed the effect of 0.1229 and its microcins in a germfree mouse model of EHEC infection.

## RESULTS

### Stx2a levels are increased upon growth in supernatants of E. coli 0.1229ΔB17.

E. coli strain 0.1229 produces two microcins: microcin B17 and the less-well-characterized Mcc1229 (15). To isolate the impact of Mcc1229, we deleted the microcin B17 operon by one-step recombination, generating 0.1229ΔB17 (15). The inactivation of both microcins was then accomplished by one-step recombination of 0.1229ΔB17 with the Δ6 PCR product, which was previously designed to remove a hypothetical protein and an ABC transporter from the Mcc1229 cluster on p0.1229_3 (15). These open reading frames (ORFs) were previously described as Hp1 and ABC based on their predicted protein products (15). In accordance with microcin nomenclature and in reference to their role in amplifying Shiga toxin, we have assigned genes in the Mcc1229 region names that begin with *mctA* (microcin involved in toxin amplification). Hp1 and ABC have been renamed *mctA* and *mctB*, respectively, and the Δ6 deletion is now referred to as Δ*mctAB* (Tables 1 and 2).

**TABLE 1:** Strains and plasmids^*a*^

Strain or plasmid	Characteristic(s)	Reference
Strains		
C600	K-12 derivative	81
MG1655	K-12 derivative	82
0.1229	O18:H1, B2 phylogroup; Amp^r^ Tet^r^	15
0.1229ΔB17::*cat*	p0.1229_2Δ*mcbABCDEFG*::*cat*; Amp^r^ Tet^r^ Cam^r^	15
0.1229ΔB17::FRT	p0.1229_2Δ*mcbABCDEFG*::FRT; Amp^r^ Tet^r^	This study
0.1229Δ*mctAB*	p0.1229_3Δ^2850–5473^::*cat*; Amp^r^ Tet^r^ Cam^r^	15
0.1229ΔB17Δ*mctAB*	p0.1229_2Δ*mcbABCDEFG*::FRT p0.1229_3Δ^2850–5473^::*cat*; Amp^r^ Tet^r^ Cam^r^	This study
0.1229Δ*fur*	Δ*fur*::*cat*; Amp^r^ Cam^r^	This study
PA2	O157:H7 *stx*_2a_	77
PA2Δ*cirA*	Δ*cirA*::*kan*; Kan^r^	This study
PA2.1	*cirA*p.Gln24ArgfsTer35	This study
PA2.2	*cirA*c.-329_-244del	This study
PA2.3	*cirA*p.Leu35GlyfsTer51	This study
EDL933	O157:H7 *stx*_1a_ *stx*_2a_	83
EDL933Δ*tonB*	Δ*tonB*::*cat*; Cam^r^	This study

Plasmids		
pKD3	Cam^r^ *oriR*γ	67
pKD4	Kan^r^ *oriR*γ	67
pKD46	P_araC_-λ recombinase; Amp^r^	67
pCP11B	FLP recombinase; Kan^r^	84
pKD46-Kan	Kan^r^	15
pBAD24	P_araC_; Amp^r^	85
pKP315	pBAD24::P_araC_-*tonB*; Amp^r^	86
pBR322	Amp^r^ Tet^r^	87
pBR322::*cirA*	Amp^r^ Tet^r^	This study
pBR322::mcc1229	pBR322::p0.1229_3^2745–7950^; Tet^r^	15
pBR322::mcc1229Δ*mctA*	pBR322::p0.1229_3^2745–7950^Δ(3084–3792); Tet^r^	This study
pBR322::mcc1229Δ*mctB*	pBR322::p0.1229_3^2745–7950^Δ(3831–5423); Tet^r^	This study
pBR322::mcc1229Δ*mctC*	pBR322::p0.1229_3^2745–7950^Δ(5426–6319); Tet^r^	This study
pBR322::*mctAB*	pBR322::p0.1229_3^2745–5425^; Tet^r^	This study
pBR322::*mctABC*	pBR322::p0.1229_3^2745–6343^; Tet^r^	This study
pBR322::*mctC*	pBR322::p0.1229_3^5426–6343^; Tet^r^	This study
pBR322::*mctCDE*	pBR322::p0.1229_3^5426-7047^; Tet^r^	This study
pBR322::*mctE*	pBR322::p0.1229_3^7047–6703^; Tet^r^	This study
pBR322::*mctD*	pBR322::p0.1229_3^6706–6320^; Tet^r^	This study
pBR322::*mctDE*	pBR322::p0.1229_3^7047–6320^; Tet^r^	This study
pRS551	Promoterless *lacZ*; Amp^r^ Kan^r^	69
pRS551::*mctA*′-*lacZ*	Amp^r^ Kan^r^	This study
pRS551::*mctB*′-*lacZ*	Amp^r^ Kan^r^	This study
pRS551::*mctC*′-*lacZ*	Amp^r^ Kan^r^	This study
pRS551::*mctE*′-*lacZ*	Amp^r^ Kan^r^	This study
pACYC184	Tet^r^ Cam^r^	88
pACYC184::P_mctA_-*mctA*	Cam^r^	This study
pACYC184::P_mctA_-*mctB*	Cam^r^	This study
pACYC184::P_mctA_-*mctC*	Cam^r^	This study

a Amp, ampicillin; Tet, tetracycline; Cam, chloramphenicol; FRT, flippase recognition target; Kan, kanamycin.

**TABLE 2:** Primers^*a*^

Primer	Sequence	*T_a_* (°C)
Δmcb_VF	GGGGCTTAAAGGGGTAGTGT	49
Δmcb_VR	CCTAACAACGCCACGACTTT	

ΔmctAB_KF	acacatttcgtacagcctttacactcggtgaattagcggccctagatgcaGTGTAGGCTGGAGCTGCTTC	67
ΔmctAB_KR	ttaaacctcatgttttgtgatatctataatctgtgctttaggtatattatCATATGAATATCCTCCTTAG	

ΔmctAB_VF	GAAGATATCGCACGCCTCTC	54.5
ΔmctAB_VR	CGCCTGTTTGGCTATATGTG	

ΔcirA_KF	gcagtatttactgaagtgaaagtccgcccggttcgccgggcatcttctcaGTGTAGGCTGGAGCTGCTTC	72
ΔcirA_KR	ctatttcttgtgcatggcctgtgttagcggtcgatgacgatggcgaaacgCATATGAATATCCTCCTTAG	

ΔcirA_VF	CCCGACGCTTATCGATCAGGG	56
ΔcirA_VR	TGGTCCGGCTTTCTGGGATG	

cirA_fwd	ggccctttcgtcttcaagaaGTTTCTCCCTTCCTTGCTAAG	57
cirA_rev	taagctgtcaaacatgagaaTCAGAAGCGATAATCCAC	

pBR322_cirF	TTCTCATGTTTGACAGCTTATC	45
pBR322_cirR	TTCTTGAAGACGAAAGGG	

pBR322_cirVF	GGGCGACACGGAAATGTTG	53
pBR322_cirVR	GCGCTAGCAGCACGCC	

Δfur_KF	aaagccaacctgcaggttggcttttctcgttcaggctggcGTGTAGGCTGGAGCTGCTTC	72
Δfur_KR	tctaatgaagtgaaccgcttagtaacaggacagattccgcCATATGAATATCCTCCTTAG	

Δfur_VF	GCCGCACGTTTGAGGAATTT	52
Δfur_VR	TTTGCCAGGGACTTGTGGTT	

pBR322_insF	GCAAAAACAGGAAGGCAAAATG	46
pBR322_insR	CTGTCAGACCAAGTTTACTC	

pBR322_mctA_F	gtatatatgagtaaacttggtctgacagGAACCTACAACACATGTGTAAAACGTCAATG	60

pBR322_mctB_R	tgccttcctgtttttgcTTTTAAACCTCATGTTTTGTG	57

pBR322_mctC_R	tgccttcctgtttttgcGGGGAAGCCCCCTTAGATTAATG	60

pBR322_mctDE_R	tgccttcctgtttttgcATATGCTTGCTTGGGAAATTC	60

pBR322_mctC_F	taaacttggtctgacagATGAATAATCTTATAAAAAAGGAAATCATAGAAAAATTTAAGAAATATAATTTC	53

pBR322_mctE_F	taaacttggtctgacagATATGCTTGCTTGGGAAATTC	58
pBR322_mctE_R	tgccttcctgtttttgcTCACACTACCTTCCTCATATC	

pBR322_mctD_F	taaacttggtctgacagGTGACTAATTTTAAATCAGACTTAAATC	54
pBR322_mctD_R	tgccttcctgtttttgcCCATTAATCTAAGGGGGC	

pBR322_insVF	TTTGCAAGCAGCAGATTACG	49
pBR322_insVR	GCCTCGTGATACGCCTATTT	

ΔmctA_F	CATAAAGCCCGTAATATAC	55
ΔmctA_R	AACACCCCCAATTATATATTTG	

ΔmctB_F	AAATGAATAATCTTATAAAAAAGGAAATC	57
ΔmctB_R	TTTATAATCCTTAAAGCCCG	

ΔmctC_F	CCATTAATCTAAGGGGGC	57
ΔmctC_R	TTTTAAACCTCATGTTTTGTG	

pACYC_PmctA_F	aacgcagtcaggcaccgtgtCGTGAGTTTTCGTTCCAC	64
pACYC_mctA_R	gaggtgccgccggcttccatTTATCTAGAATTACATGAGCAC	

PmctA_mctB_R	taaaattcaaAACACCCCCAATTATATATTTG	47

PmctA_mctB_F	tgggggtgttTTGAATTTTATTGAAAAGTACATCATC	54
pACYC_mctB_R	gaggtgccgccggcttccatTTAAACCTCATGTTTTGTGATATC	

PmctA_mctC_R	gattattcatAACACCCCCAATTATATATTTG	47

PmctA_mctC_F	tgggggtgttATGAATAATCTTATAAAAAAGGAAATCATAG	52
pACYC_mctC_R	gaggtgccgccggcttccatTTATATATGTTCAAGTTTTATTTTTTTATAGC	

pACYC_dTc_F	ATGGAAGCCGGCGGCACC	58
pACYC_dTc_R	ACACGGTGCCTGACTGCG	

pACYC_VF	GCAAGAGATTACGCGCAGAC	53
pACYC_VR	TAACCAGTAAGGCAACCCCG	

pRS551_mctA_F	GAATTCTATAACCATTAAAAAACTTGATTACTATCTC	47
pRS551_mctA_R	CCCGGGATCCTTAGAAGAACATCATC	

pRS551_mctB_F	GAATTCCAAAAGAATCCATATCCAG	47
pRS551_mctB_R	CCCGGGTAAGCAGGATCCTATTTCTCCTATTGAATC	

pRS551_mctC_F	TAAGCAGAATTCGCTACACAGATTTAAG	49
pRS551_mctC_R	TAAGCAGGATCCATAGTGCAATATATC	

pRS551_mctE_F	TAAGCAGAATTCGCTGCATAGCTATGCATG	55
pRS551_mctE_R	TAAGCAGGATCCTATGACTGGGATTACTCT	

pRS551_VF	TGCCAGGAATTGGGGATC	51
pRS551_VR	GTTTTCCCAGTCACGACGTT	

qPCR_rrsH_F	CGATGCAACGCGAAGAACCT	60
qPCR_rrsH_R	CCGGACCGCTGGCAACAAA	

qPCR_mctA_3393F (Afwd)	TCTTGGTGCTTACCTCCACCA	60
qPCR_mctA_3556R	TTGCGACAGTTTCATGACCCA	

qPCR_mctB_4389F (Bfwd)	GCACTTAGCTCCAAATTCGC	60
qPCR_mctB_4567R (Brev)	GCGGAGCTGATACCAAACAG	

qPCR_mctC_5589F (Cfwd)	TGGCAAATGACAACTTTCCCG	60
qPCR_mctC_5715R (Crev)	GCGCCATCACGTAAGCATTT	

qPCR_mctD_6365F	AAACTGCATTTTCCATCCACCA	60
qPCR_mctD_6541R (Drev)	CGTCCAGCGAGGATTTTACC	

a Ta, annealing temperature. In primer sequences, lowercase letters indicate homology to the chromosome (for KF/KR knockout primers) or overlapping regions (for Gibson assembly primers). Underlined sequences represent recognition sites for restriction enzymes.

**FIG 1:**
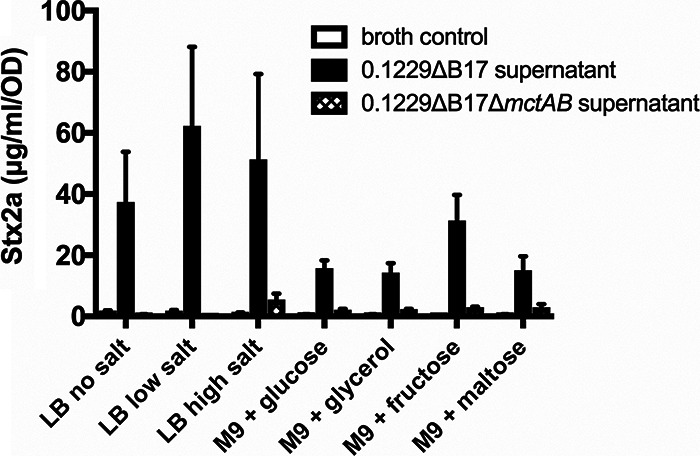
Stx2a levels are amplified by culture supernatants from Mcc1229-producing strains. Cell-free supernatants of E. coli 0.1229ΔB17::FRT grown in various media were used to culture E. coli O157:H7 strain PA2. Stx2a levels were measured by an R-ELISA and normalized to the OD_620_ of each culture; the means and standard errors of the means (SEM) are reported (minimum *n* = 3). M9 medium was supplemented with 0.1% Casamino Acids, 0.005% thiamine, and 0.4% of the indicated carbon source. Cultures grown in the spent supernatants of 0.1229ΔB17::FRT are indicated by solid bars. Cultures grown in the spent supernatants of the double microcin mutant 0.1229ΔB17::FRTΔ*mctAB*::*cat* are given as crosshatched bars. For comparison, PA2 was grown in fresh medium of the same composition, as indicated by empty bars. All such “broth control” cultures yielded less than 5 μg/ml/OD Stx2a.

To evaluate the effect of culture conditions on the production of Mcc1229, E. coli 0.1229ΔB17 was grown in various liquid media. These included lysogeny broth (LB) with 0%, 0.5%, and 1% NaCl (no, low, and high salt, respectively) and M9 medium supplemented with Casamino Acids, thiamine, and 0.4% glucose, glycerol, maltose, or fructose. Stx2a amplification by Mcc1229 was determined by culturing the *stx*_2a_^+^ E. coli O157:H7 strain PA2 in spent supernatants. All spent supernatants from 0.1229ΔB17 supported the growth of PA2 and increased Stx2a levels above those in broth alone (Fig. 1). The differences in Stx2a amplification among the media were not statistically significant (Fig. 1). Amplification was dependent on microcin production, as demonstrated by the supernatants of 0.1229ΔB17Δ*mctAB*. This strain, which produced neither MccB17 nor Mcc1229, induced minimal levels of Stx2a that were statistically equivalent to those of the broth controls (*P* = 0.9789) (Fig. 1).

### Iron suppresses Stx2a-amplifying activity.

**FIG 2:**
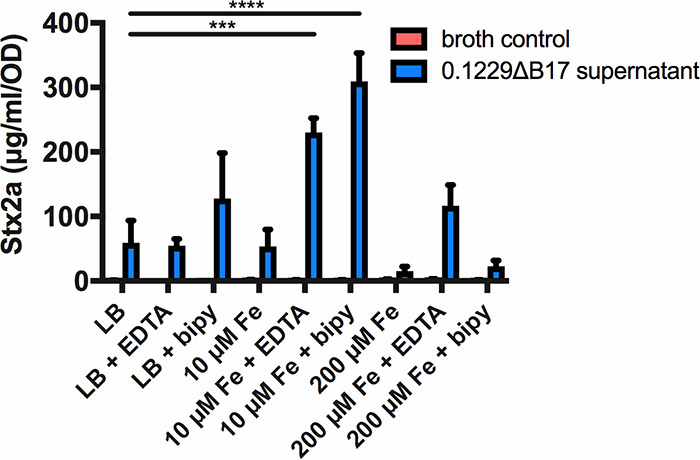
Stx2a levels are diminished when cells are grown in supernatants from high-iron media. Cell-free supernatants of E. coli 0.1229ΔB17::FRT grown in various media were used to culture E. coli O157:H7 strain PA2. For comparison, PA2 was grown in fresh medium of the same composition, as indicated by “broth control.” Stx2a levels were measured by an R-ELISA and normalized to the optical density of each culture; the means and SEM are reported (*n* = 3 for all except 10 μM Fe cultures, in which *n* = 2). The metal chelators EDTA and 2,2′-bipyridyl (bipy) were added to media at 0.2 mM. Statistical significance was determined by two-way analysis of variance (ANOVA) and Sidak’s multiple-comparison test, assigning LB as the standard for the broth control and 0.1229ΔB17::FRT supernatant groups (***, *P* < 0.001; ****, *P* < 0.0001).

To further investigate the conditions influencing Mcc1229 expression, we added the metal-chelating agent EDTA or 2,2′-bipyridyl (bipy) to cultures of 0.1229ΔB17 grown in LB with high salt. EDTA chelates a variety of divalent cations, while bipyridyl has a high affinity for iron. Supernatants from 0.1229ΔB17 cultures grown in LB plus bipyridyl amplified Stx2a levels beyond those from LB alone (Fig. 2). In the presence of low concentrations (10 μM) of ferric chloride, EDTA- and bipyridyl-supplemented supernatants still significantly amplified Stx2a above unsupplemented LB supernatant levels (Fig. 2). When FeCl_3_ levels were increased to 200 μM, equimolar to EDTA or bipyridyl, the Stx2a-amplifying effect of these supernatants was suppressed (Fig. 2). In other words, excess iron negated the impact of bipyridyl on Mcc1229. In contrast, the addition of 200 μM CaCl_2_, MgCl_2_, or MnCl_2_ to 0.1229ΔB17 cultures did not reduce their Stx2a-amplifying activity (data not shown), suggesting that this effect was specific to FeCl_3_.

### *mctA*, *mctB*, and *mctC* ORFs are required for Stx2a amplification.

**FIG 3:**
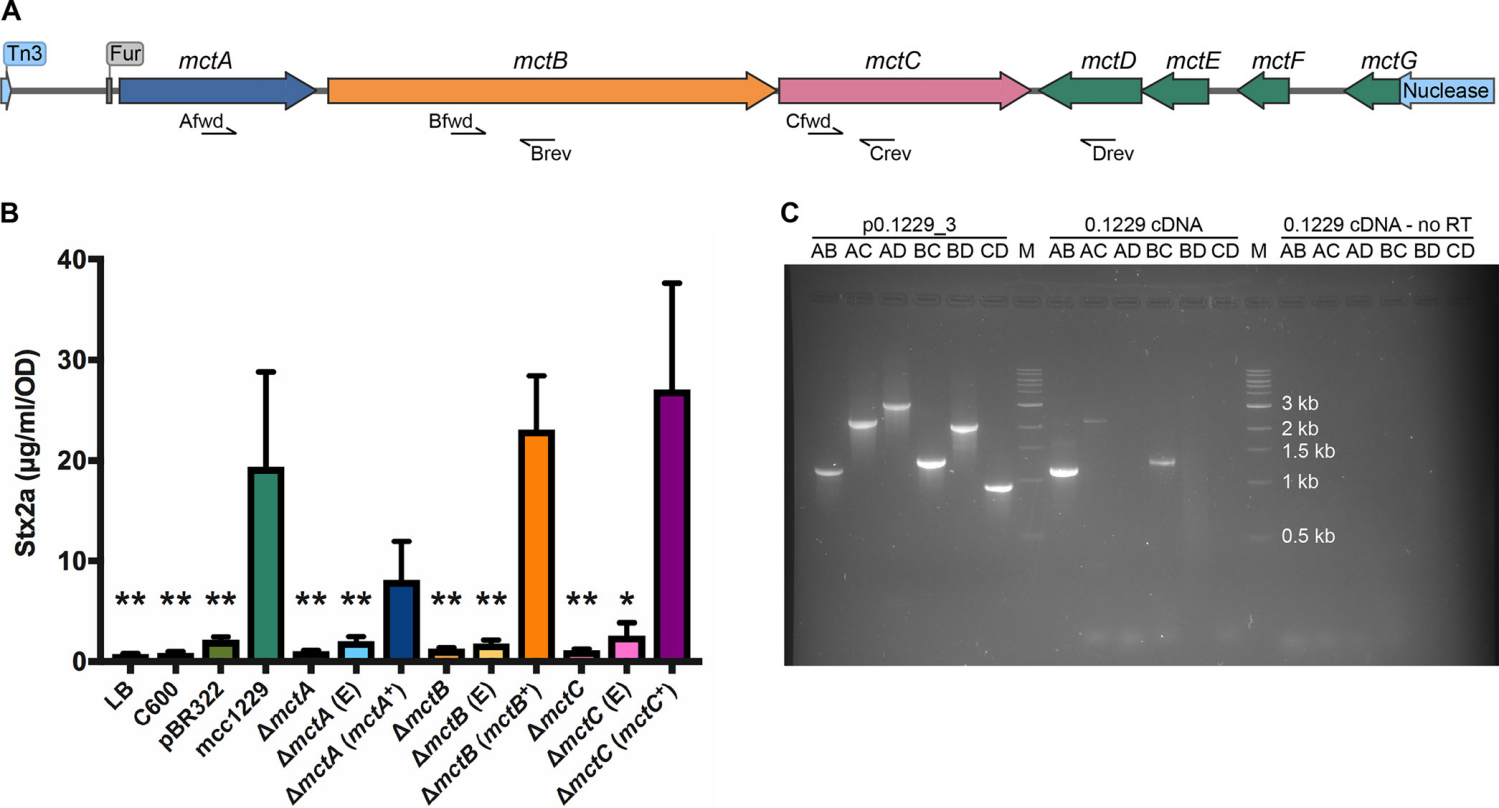
A 5.2-kb region of p0.1229_3 sufficient for Stx2a amplification harbors seven putative open reading frames. (A) Annotation of p0.1229_3 was performed by NCBI’s Prokaryotic Genomes Automatic Annotation Pipeline (PGAAP) as reported previously (15, 78). A Fur site preceding *mctA* was identified by the matrix-scan algorithm at the RSAT Prokaryotes Web server (75, 79). The map diagram was generated by SnapGene software (from GSL Biotech). (B) The mcc1229 region of p0.1229_3 was cloned into pBR322 and was sufficient to amplify Stx2a. Supernatants from the C600 strain alone or from the empty vector pBR322 did not amplify Stx2a. Deletions of *mctA*, *mctB*, and *mctC* abolished Stx2a-amplifying activity and could be complemented in *trans*. “E” denotes the empty complementation vector, pACYC124. Stx2a expression of PA2 exposed to filtered culture supernatants was determined by an ELISA as described in the text. Values that differed significantly from the mcc1229 supernatant are marked with asterisks (*, *P* < 0.05; **, *P* < 0.01). Statistical analysis was performed by one-way ANOVA with Dunnett’s multiple-comparison test. (C) *mctABC* form a transcriptional unit. Total RNA was extracted from 16-h-grown cultures of E. coli 0.1229 and converted to cDNA. PCR primers internal to the *mctA*, *mctB*, and *mctC* genes, depicted as half-arrows in panel A, amplified fragments indicative of a polycistronic transcript. Plasmid DNA (p0.1229_3) and a cDNA reaction without reverse transcriptase (RT) were used as positive and negative controls, respectively. “M” denotes the molecular marker, NEB’s 1-kb DNA ladder.

Previous work demonstrated that the p0.1229_3 plasmid, and specifically a 5.2-kb region therein, was sufficient to amplify Stx2a (15). This region was moved into the medium-copy-number pBR322 vector, replacing the *bla* gene (15). The resulting construct is termed pBR322::mcc1229. The mcc1229 region is predicted to encode three hypothetical proteins (MctA, MctF, and MctG), an ABC transporter (MctB), a cupin domain protein (MctC), and two domain of unknown function (DUF) proteins (MctD and MctE) (Fig. 3A). Each of these was previously deleted by one-step recombination in the native 0.1229 strain, inserting a *cat* marker in its place on p0.1229_3. *mctA*, *mctB*, *mctC*, and *mctF* mutants were significantly impaired for Stx2a amplification (15). To avoid any potential polar effects of the *cat* insertions, here, we constructed markerless in-frame deletions of the same ORFs in the pBR322::mcc1229 clone instead. When the *mctA*, *mctB*, and *mctC* ORFs were deleted from this region, the resulting constructs no longer amplified Stx2a (Fig. 3B). Supernatants from C600 (pBR322::mcc1229Δ*mctA*), C600 (pBR322::mcc1229Δ*mctB*), and C600 (pBR322::mcc1229Δ*mctC*) were indistinguishable from an empty vector control (pBR322) (Fig. 3B). Stx2a amplification was restored when the deletion mutants were complemented with plasmid copies of the given ORF (*mctA*^+^, *mctB*^+^, and *mctC*^+^) (Fig. 3B). PCR analysis of cDNA prepared from total RNA of 0.1229 cultures indicated that *mctA*, *mctB*, and *mctC* comprise an operon (Fig. 3C).

No mutants were obtained when *mctD* or *mctE* was targeted for in-frame deletion, suggesting that these genes play an essential role in the stability of the pBR322::mcc1229 construct. C600 (pBR322::mcc1229Δ*mctF*) and C600 (pBR322::mcc1229Δ*mctG*) were not diminished in their ability to amplify Stx2a (see Fig. S1 in the supplemental material).

### p0.1229_3 ORFs are in the Fur regulon.

**FIG 4:**
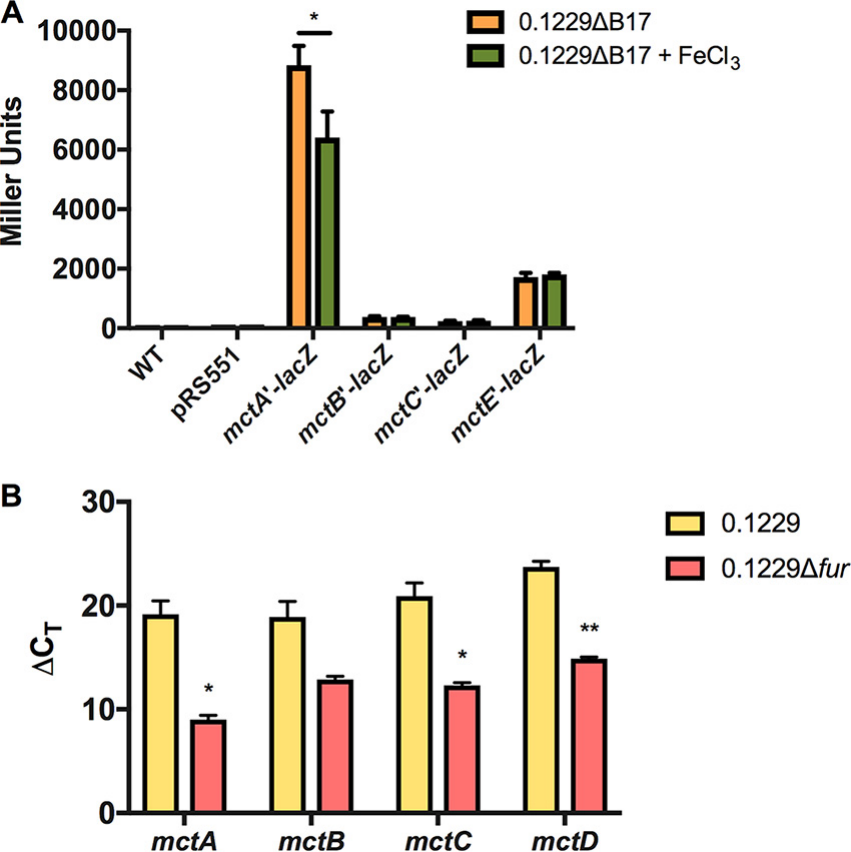
Mcc1229 transcription is iron-regulated. (A) A region upstream of the *mctA* ORF containing a putative Fur binding site was ligated into pRS551 and successfully promoted the transcription of *lacZ*. Promoter regions upstream of the *mctB*, *mctC*, and *mctE* ORFs were also tested in this manner. The transcriptional activity of the *mctA* promoter decreased when the medium was supplemented with 200 μM FeCl_3_. Significance was determined by two-way ANOVA with Sidak’s multiple-comparison test. (B) RNA was extracted from 16-h-grown LB cultures of 0.1229 and 0.1229Δ*fur*::*cat*, converted to cDNA, and probed by qPCR for the *mctA*, *mctB*, *mctC*, and *mctD* ORFs. Gene expression was determined by the Δ*C_T_* method using the ribosomal gene *rrsH* as an internal control (80). Taking the differences of Δ*C_T_*_,WT_ and Δ*C_T_*_,Δ_*_fur_* gives ΔΔ*C_T_* values of 10.14, 6.04, 8.60, and 8.85 for *mctA*, *mctB*, *mctC*, and *mctD*, respectively. All *mct* genes examined were consistently upregulated in the Δ*fur*::*cat* strain. Statistical significance was determined by Student’s *t* test on the Δ*C_T_* values for each gene (*, *P* < 0.05; **, *P* < 0.01).

Sequence analysis of the Stx2a-amplifying region on p0.1229_3 revealed a putative Fur binding site (ataAATGATAActATTcTC, where uppercase letters indicate identity to the consensus [43]) upstream of the *mctA* open reading frame (Fig. 3A). The region upstream of *mctA* was ligated into pRS551, and it successfully promoted the transcription of *lacZ* when expressed in 0.1229ΔB17 (Fig. 4A). Promoter regions upstream of the *mctB*, *mctC*, and *mctE* ORFs were also verified in this manner (Fig. 4A). The transcriptional activity of the *mctA* promoter decreased when the medium was supplemented with ferric chloride (Fig. 4A), suggesting that MctA was regulated by iron. These data were supported by transcriptional analyses of 0.1229 and 0.1229Δ*fur*. Quantitative real-time PCR (qRT-PCR) targeting *mctA*, *mctB*, *mctC*, and *mctD* regions indicated that the expression of each gene was increased in the *fur* mutant (Fig. 4B), consistent with a model in which the Fe-Fur complex repressed the transcription of the microcin.

### CirA is the outer membrane receptor for Mcc1229.

**FIG 5:**
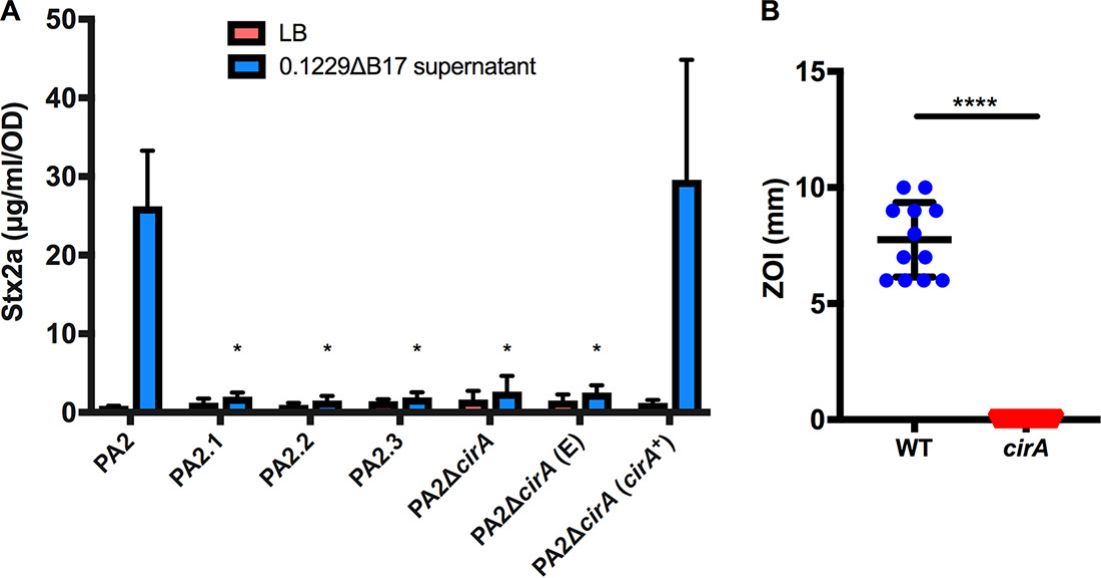
CirA is the outer membrane receptor for Mcc1229. (A) Spontaneous mutants of PA2 were resistant to inhibition by E. coli 0.1229ΔB17::*cat*. Colonies were isolated from within the zones of clearing and subjected to whole-genome sequencing to identify the source of Mcc1229 resistance. Multiple independent mutants (PA2.1, PA2.2, and PA2.3) had mutations in CirA. A Δ*cirA*::*kan* mutant of PA2 is resistant to Mcc1229, and when *cirA* mutants are grown in the spent supernatants of E. coli 0.1229ΔB17::*cat*, they are insensitive to Stx amplification. Sensitivity is restored by complementation with pBR322::*cirA* (*cirA*^+^) but not with the pBR322 empty vector (“E”). Asterisks mark significant differences between a given supernatant sample and the PA2 wild type (two-way ANOVA and Dunnett’s multiple-comparison test). (B) Colicin indicator strains confirmed that CirA was the Mcc1229 receptor. NCTC 50154 (WT) and NCTC 50157 (*cirA*) were tested in agar overlay assays. The resulting zones of inhibition (ZOI) were significantly different (*P* < 0.0001 by Student’s *t* test).

To identify the receptor for Mcc1229, we investigated its entry into target cells in several ways. First, in an agar overlay assay, we showed that Mcc1229 inhibited the susceptible E. coli O157:H7 strain PA2, creating a zone of clearing around Mcc1229-producing colonies (Fig. S2). Spontaneous Mcc1229-resistant mutants of PA2 that grew within the zone of inhibition (ZOI) were then subjected to whole-genome sequencing. Three independent colonies revealed mutations predicted to affect the expression of *cirA*, which encodes a catecholate siderophore receptor: two contained frameshift mutations in the *cirA* ORF that introduced a premature stop codon, and one carried an 86-bp deletion directly upstream of the *cirA* start codon (Table 1). These isolates (PA2.1, PA2.2, and PA2.3) were insensitive to Stx2a amplification by 0.1229ΔB17 supernatants (Fig. 5A). Second, a targeted deletion of *cirA* in PA2 by one-step recombination (Δ*cirA*) was also resistant to Mcc1229-mediated Stx2a amplification (Fig. 5A). Sensitivity was restored by complementation with the medium-copy-number plasmid pBR322::*cirA* (*cirA*^+^) (Fig. 5A). Finally, *cirA* was confirmed as the Mcc1229 receptor using a set of indicator strains bearing mutations in known colicin receptors (44). While a wild-type (WT) indicator strain was susceptible to Mcc1229 inhibition in the agar overlay method, a *cirA* mutant was resistant to this microcin (Fig. 5B).

### Mcc1229 entry requires TonB.

**FIG 6:**
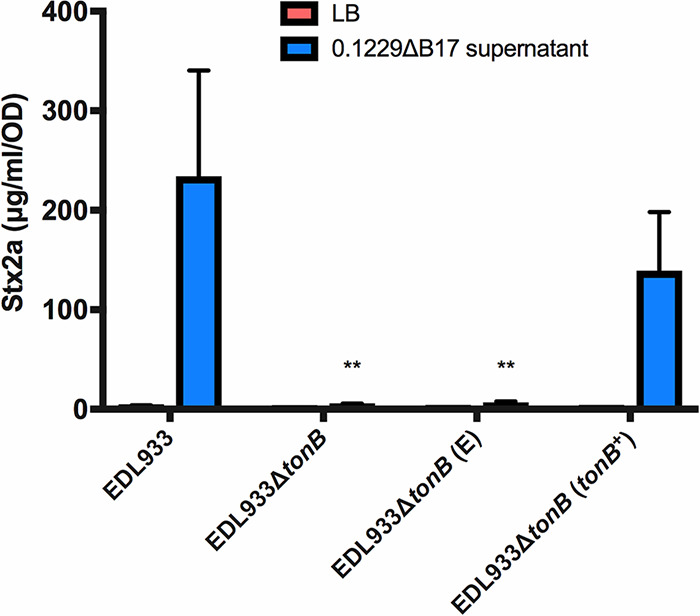
TonB is required for Mcc1229 activity. The periplasmic energy-transducing protein TonB was deleted from E. coli O157:H7 strain EDL933 by one-step recombination. The resulting mutant does not increase Stx expression in response to spent supernatants containing Mcc1229. Activity is restored upon complementation with a plasmid copy of *tonB*, carried on pKP315 (*tonB*^+^). EDL933Δ*tonB* (E) represents the empty vector strain, which carries pBAD24. Because *tonB* is under P_araC_ control on pKP315, all strains in this experiment were grown in the presence of arabinose. Asterisks mark significant differences between a given supernatant sample and the EDL933 wild type (two-way ANOVA and Dunnett’s multiple-comparison test).

CirA is a known TonB-dependent transporter, and the import of CirA-dependent colicins requires the activity of TonB in the periplasm (45). Previous data also implicated TonB in SOS induction by Mcc1229 in a reporter strain (15). We next sought to inactivate *tonB* in E. coli O157:H7 to determine its role in Stx2a amplification by Mcc1229. Attempts to delete *tonB* in the PA2 background by various methods were unsuccessful. *tonB* was instead deleted by one-step recombination in EDL933, a well-characterized *stx*_1a_^+^ *stx*_2a_^+^ O157:H7 strain (46). A Δ*tonB* mutant did not amplify Stx2a in response to supernatants containing Mcc1229 (Fig. 6). When complemented with a plasmid copy of *tonB* (pKP315), the strain behaved like the wild type (Fig. 6). An empty vector (“E”) (pBAD24) did not restore the phenotype (Fig. 6). These data indicated that both CirA and TonB were necessary for Stx2a amplification by Mcc1229.

### Immunity to Mcc1229 is mediated by the *mctD-mctE* region of p0.1229_3.

**FIG 7:**
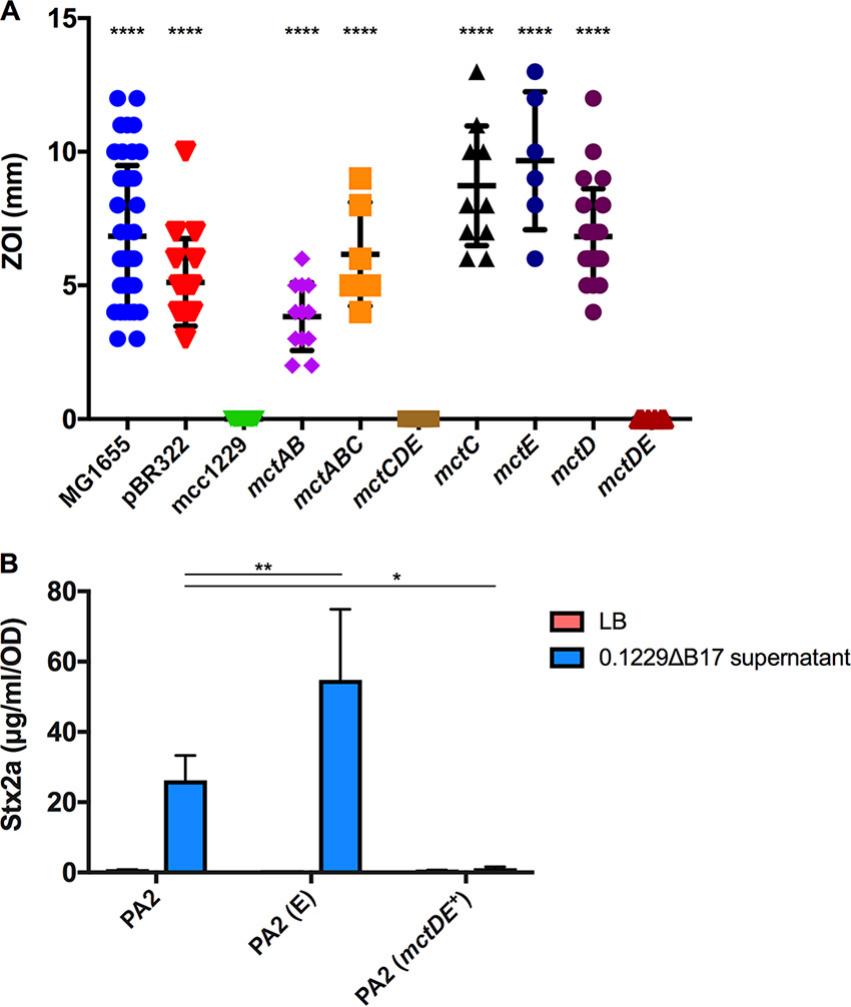
The *mctD*-*mctE* region is sufficient to confer immunity to Mcc1229. (A) Two p0.1229_3 ORFs annotated as proteins with domains of unknown function were cloned into pBR322 and transformed into E. coli strain MG1655. MG1655 carrying an empty vector (pBR322) is sensitive to Mcc1229 produced by E. coli 0.1229ΔB17::*cat*. MG1655 carrying pBR322::*mctDE* is fully resistant to the microcin. Strains that differed significantly from the full-length construct (mcc1229) are marked with asterisks (*P* < 0.0001 by one-way ANOVA with Dunnett’s multiple-comparison test). (B) Transformants of PA2 carrying pBR322::*mctDE* (*mctDE*^+^) do not increase Stx expression when grown in spent supernatants of E. coli 0.1229ΔB17::*cat*. PA2 (E) represents the empty vector strain PA2 (pBR322). Asterisks mark significant differences between a given supernatant sample and the PA2 wild type (*, *P* < 0.05; **, *P* < 0.01 [by two-way ANOVA and Dunnett’s multiple-comparison test]).

The lethality of colicins and microcins necessitates a mechanism of protection for the producing cell. By cloning progressively smaller fragments of p0.1229_3 into pBR322, we identified a region of the plasmid that was sufficient to confer immunity to Mcc1229. Two adjacent ORFs, *mctD* and *mctE*, each predicted to encode a DUF-containing protein, protected MG1655 from Mcc1229-mediated killing in an agar overlay assay (Fig. 7A). Vectors containing either of the single ORFs were not protective (Fig. 7A). When the pBR322::*mctDE* construct was transformed into PA2, PA2 became insensitive to the 0.1229ΔB17 supernatant, and Stx2a production was minimal (Fig. 7B).

### Mcc1229 is expressed *in vivo* but is not required for suppression of PA2.

**FIG 8:**
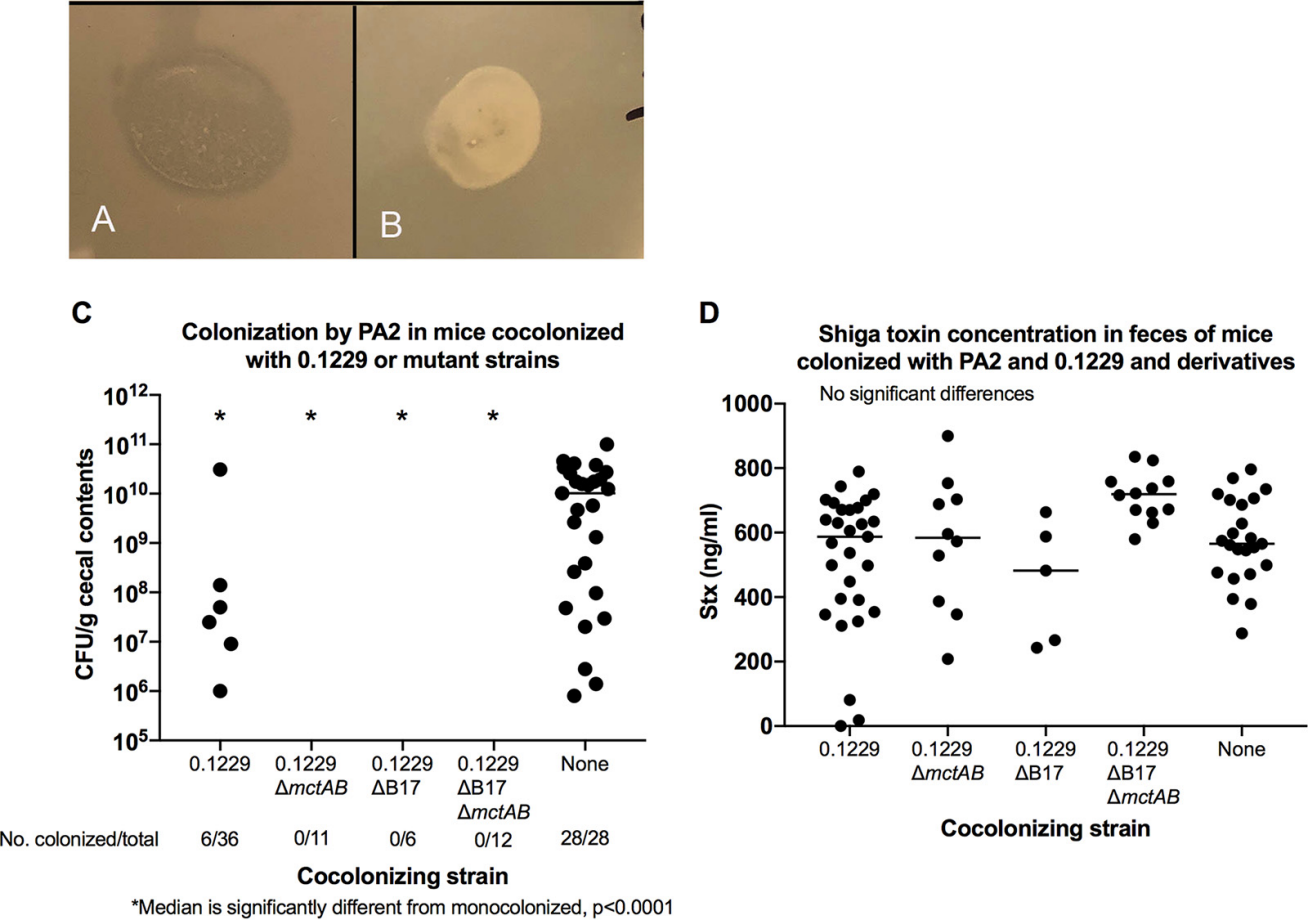
(A and B) Soft agar containing a suspension of PA2 was overlaid onto LB and allowed to solidify, and fecal supernatants from infected mice were spotted onto the overlay. Plates were incubated overnight at 37°C. (A) Fecal supernatants from mice cocolonized with 0.1229 and PA2 prevented the growth of PA2, resulting in a zone of clearing in the soft agar. (B) Supernatants from mice colonized with 0.1229ΔB17::FRTΔ*mctAB*::*cat* did not affect PA2 growth. (C) PA2 colonization in cocolonized mice. PA2 colonization was detectable in only 17% of mice cocolonized by wild-type 0.1229 and in none of the mice colonized by 0.1229 Δ*mctAB*::*cat*, 0.1229ΔB17::FRT, or 0.1229ΔB17::FRT Δ*mctAB*::*cat*. In contrast, 100% of mice inoculated with PA2 alone became colonized (also see Table 3). (D) Low colonization levels did not affect Stx production. Stx was detected in all mouse groups, and concentrations ranged from 18 to 900 ng/mL. There were no differences between groups.

To determine the effect of microcins *in vivo*, we colonized germfree mice with E. coli 0.1229 and its derivatives and collected fecal samples at 1 day postinfection (p.i.). After suspending fecal samples in LB, the samples were centrifuged to pellet the solid matter, and the supernatant was spotted on top of a suspension of the PA2 test strain. The supernatants from mice infected with 0.1229 inhibited the growth of PA2, but those from mice infected with an Mcc1229 knockout strain had no effect (Fig. 8A and B).

The role of Mcc1229 in the germfree mouse model of EHEC was investigated by the sequential inoculation of 0.1229 and PA2. Mice were first infected with 0.1229 or its derivatives and then challenged with PA2 7 days later. Monoinfections with 0.1229 or PA2 served as controls. PA2 alone was able to colonize at concentrations of between 10^8^ and 10^10^ CFU/g and caused symptoms consistent with Stx-mediated disease, including colitis and acute kidney injury (47, 48). When mice were colonized with 0.1229 prior to the introduction of PA2, PA2 colonization was rarely detected. PA2 was recovered from the cecal contents of only 6 of 65 mice coinfected with 0.1229 or its derivatives (Fig. 8C and Table 3). This effect did not require Mcc1229 or MccB17, however, as the single and double microcin mutants of 0.1229 were capable of suppressing PA2 equivalently to the wild type (Fig. 8C and Table 3). Colonization suppression was not protective against disease, as PA2 was still lethal to coinfected mice, and Stx was detected in fecal samples from all groups (Fig. 8D and Table 4). This likely indicates that PA2 was present at some time during infection but was either lost or suppressed below the limit of detection.

**TABLE 3:** Colonization by PA2 in mice cocolonized by 0.1229 and derivatives

Cocolonizing strain	No. of mice in group	No. of mice colonized	% of mice colonized
0.1229	36	6	17
0.1229Δ*mctAB*	11	0	0
0.1229ΔB17	6	0	0
0.1229ΔB17Δ*mctAB*	12	0	0
None	28	28	100

**TABLE 4:** Clinical illness due to PA2 in mice cocolonized by 0.1229 or its derivatives

Infection	No. moribund or dead mice/total no. of mice by 7 days p.i.	% of moribund or dead mice
PA2 alone	4/28	14
0.1229 + PA2	14/36	39
0.1229Δ*mctAB* + PA2	2/11	18
0.1229ΔB17 + PA2	2/6	33
0.1229ΔB17Δ*mctAB* + PA2	0/12	0

## DISCUSSION

A putative microcin from the human E. coli isolate 0.1229 was previously shown to induce the SOS response and Stx expression in target strains (15). Here, we have confirmed the activity of this microcin (Mcc1229), isolated its activity from that of a second microcin encoded by 0.1229 (MccB17), and further characterized its production, regulation, and effects. Like several other colicins and microcins, Mcc1229 uses the CirA siderophore receptor (Fig. 5) and the TonB complex (Fig. 6) for entry into a target cell. CirA was first identified as the colicin I receptor and is also used by colicin/microcin V (33, 49). In the producing strain, evidence suggests that Mcc1229 requires the *mctABC* operon of plasmid p0.1229_3 for activity (Fig. 3). Open reading frames similar to *mctA*, with cysteine-rich C-terminal regions and cognate ABC transporters, are also consistent with typical microcin operons (50).

The functional contributions of the cupin-like *mctC* and DUF-containing *mctD*-*mctE* ORFs in the Mcc1229 cluster have not yet been elucidated. In our system, the *mctDE* region conferred immunity to Mcc1229 killing and Stx amplification (Fig. 7), and we were unable to generate in-frame deletions of these ORFs. It is possible that previous mutants constructed by one-step recombination (15) retained wild-type plasmid copies or acquired secondary mutations that masked this effect. Still, it is not clear whether the DUF-containing proteins encoded by *mctD* and *mctE* directly interact with the microcin. Current Pfam records indicate that the DUF2164 domain present in MctE is found in 804 protein sequences in 715 species, but it is not associated with a clan or superfamily (51). DUF4440, which is found in MctD, belongs to a family in the nuclear transport factor 2 (NTF2) clan, which includes numerous proteins with enzymatic and nonenzymatic functions (52). Some proteins with NTF2-like folds are known to provide immunity to bacterial toxins, but their sequences (Pfam PF15655) are diverse and dissimilar to the DUF4440 domain in MctD (53). Proteins with DUF4440 and/or NTF2-like domains have also been shown to operate in polyketide biosynthesis pathways, where they are involved in catalyzing the formation of natural products (54, 55). Some proteins with cupin domains have enzymatic activity (56, 57), so it is possible that the p0.1229_3 MctC is involved in the processing or modification of Mcc1229. Contrary to previous reports, we found that neither MctF nor MctG was essential for microcin activity (see Fig. S1 in the supplemental material). Based on their homology to MbeD and MbeB family mobilization/exclusion proteins, we hypothesize that deletions of the *mctF* and *mctG* ORFs may have altered plasmid maintenance or copy number.

Beyond its cellular export and import, the observed Fe-Fur regulation of Mcc1229 further supports its classification as a microcin. Mcc1229’s amplification of Stx was increased in the presence of chelating agents, and this effect could be reversed by the addition of iron specifically (Fig. 2). Moreover, the expression levels of the *mctA*, *mctB*, *mctC*, and *mctD* genes were increased in a Δ*fur* background (Fig. 4B). Taken together, these data likely indicate that Mcc1229 is transcriptionally repressed by the canonical Fe-Fur complex (58). A similar pattern is seen in the regulation of microcin E492 in Klebsiella pneumoniae (29). Like the site upstream of *mceX* in the MccE492 operon, the putative Fur box upstream of *mctA* is 68% (13/19 nucleotides [nt]) identical to the consensus Fur sequence described for E. coli (59, 60). Fur-regulated microcins may provide a competitive advantage for E. coli strains *in vivo* where iron availability is restricted (61, 62).

In our study, the microcin Mcc1229 was produced *in vivo* but had no effect on EHEC colonization or disease (Fig. 8). Nevertheless, we observed a striking example of suppression by E. coli 0.1229 in which PA2 was rarely if ever recovered from coinfections. Most other E. coli strains do not suppress EHEC to the same extent, although there is precedent for colonization suppression by the probiotic strain Nissle 1917 (63, 64). Intriguingly, both Nissle and 0.1229 belong to sequence type 73 (ST73), a lineage frequently isolated from extraintestinal pathogenic E. coli (ExPEC) infections (65). ST73 strains carry a broad assortment of virulence factors, including many genes for adherence and iron acquisition, that could provide a selective advantage over competitors (66). Ongoing studies may determine whether colonization resistance is a trait that is common to ST73.

Interactions with the microbiome can alter the virulence of EHEC in numerous ways. Understanding these effects will help predict the unique pathogenicity and disease outcomes of a given infection. Here, we have expanded upon the attributes of Mcc1229, a new E. coli microcin that induces the SOS response and amplifies Stx2a expression *in vitro*. When characterizing the interplay of Mcc1229 and EHEC *in vivo*, however, we found that microcin activity was not a significant contributor to EHEC virulence or colonization efficiency. This discrepancy highlights the need for additional research regarding the dynamics of bacteriocin expression in the intestinal environment. The regulation, stability, and activity spectrum of bacteriocins all influence their physiological role, as do external factors such as inflammation and nutrient availability. Although Mcc1229 can be unified with other microcins based on the cellular factors described in this work, its actual ecological impact was not apparent from our germfree mouse model and awaits further clarification.

## MATERIALS AND METHODS

### Bacterial strains and culture conditions.

E. coli strains were routinely grown in lysogeny broth (LB) (10 g/L tryptone, 5 g/L yeast extract, 10 g/L NaCl) at 37°C and maintained in 20% glycerol at −80°C. Minimal medium (M9) was formulated with 12.8 g/L Na_2_HPO_4_·7H_2_O, 3 g/L KH_2_PO_4_, 0.5 g/L NaCl, 1 g/L NH_4_Cl, 2 mM MgSO_4_, and 0.1 mM CaCl_2_. M9 medium was supplemented with 0.1% Casamino Acids, 0.005% thiamine, and 0.4% of the desired carbon source. Mueller-Hinton (MH) agar was prepared according to the manufacturer’s instructions. EDTA, 2,2′-bipyridyl, FeCl_3_, CaCl_2_, MgCl_2_, and MnCl_2_ were added to media at 0.2 mM. The following antibiotics were used: ampicillin at 50 μg/mL, chloramphenicol at 12.5 μg/mL, kanamycin at 25 μg/mL, and tetracycline at 10 μg/mL. All medium components were purchased from BD Difco (Franklin Lakes, NJ), and all enzymes were purchased from New England BioLabs (NEB) (Ipswich, MA), unless otherwise noted.

### One-step recombination.

E. coli knockouts were constructed according to the protocol of Datsenko and Wanner (67). Primers incorporating 40 bp immediately upstream and downstream of the gene of interest were used to amplify the *cat* cassette from pKD3 or the *kan* cassette from pKD4 (Tables 1 and 2). The target strain was first transformed with pKD46, grown to mid-log phase, and then induced with 0.02 M l-arabinose for 1 h. Cells were washed with cold water and 10% glycerol and electroporated with the *cat* or *kan* PCR product using a GenePulser II instrument (2.5 kV, 0.2-cm-gap cuvettes; Bio-Rad, Hercules, CA). Transformants were verified by colony PCR with primers approximately 200 bp up- and downstream of the gene of interest, and the site of the insertion was confirmed by Sanger sequencing (Tables 1 and 2). Mutants were complemented with a plasmid copy of the gene of interest and cloned into the medium-copy-number vector pBR322 by Gibson assembly (68). Assembly primers were designed using NEBuilder (NEB) (Table 2). Amplicons were purified with the QIAquick cleanup kit (Qiagen, Germantown, MD) and assembled with the Gibson assembly cloning kit according to the manufacturers’ instructions. Assembly junctions were likewise confirmed by colony PCR and Sanger sequencing (Tables 1 and 2).

Because multiple efforts to inactivate *tonB* in PA2 by one-step recombination were unsuccessful, we generated a Δ*tonB*::*cat* mutant in the EDL933 background. This mutant was complemented by pKP315, kindly provided by Kathleen Postle, which carries an arabinose-inducible copy of *tonB* on a pBAD24 backbone. l-Arabinose was added to EDL933 cultures at 0.3%.

### *lacZ* fusions.

Transcriptional activity was measured by fusing selected p0.1229_3 fragments to a promoterless *lacZ* gene in the pRS551 vector (69). Fragments were amplified from p0.1229_3 using the indicated primers (Tables 1 and 2) and digested with EcoRI-HF and BamHI-HF enzymes. The products were cleaned up using the QIAquick kit and ligated into an EcoRI-BamHI digest of pRS551. Ligation mixtures were transformed into chemically competent DH5α cells (New England BioLabs) and verified by miniprep and restriction digests. The constructs were then electroporated into E. coli 0.1229ΔB17. Reporter strains were cultured in LB, with shaking at 37°C, and grown to mid-logarithmic phase. Cells were then harvested and suspended in Z buffer (0.06 M Na_2_HPO_4_·7H_2_O, 0.04 M NaH_2_PO_4_·H_2_O, 0.01 M KCl, 0.001 M MgSO_4_·7H_2_O, 0.05 M β-mercaptoethanol [pH 7]). LacZ activity was measured by the hydrolysis of *o*‐nitrophenyl‐β‐d‐galactoside according to the method of Miller (70).

### qPCR.

RNA was extracted from 16-h-grown LB cultures of 0.1229 and 0.1229Δ*fur*::*cat* using the Qiagen RNeasy minikit according to the manufacturer’s instructions. Genomic DNA was removed by digestion with RQ1 RNase-free DNase (Promega, Madison, WI), and RNA was converted to cDNA using the SuperScript IV kit (Thermo Fisher). The expression of the *mctA*, *mctB*, *mctC*, and *mctD* genes was quantified in 25-μL reaction mixtures using PerfeCTa SYBR green FastMix (Quantabio, Beverly, MA) and 200 nM quantitative PCR (qPCR) primers (Table 2) on a QuantStudio3 instrument (Thermo Fisher, Waltham, MA). To validate the efficiency (>95%) of each primer pair, its target was amplified from genomic DNA and purified using a spin column cleanup kit (Dot Scientific Inc., Burton, MI). The concentration of this product was measured by spectrophotometry (NanoDrop 1000; Thermo Fisher), and 10-fold dilutions ranging from 10^−2^ to 10^−7^ ng/μL were used as the templates in qPCR. A standard curve was constructed from the resulting threshold cycle (*C_T_*) values. Differences in gene expression between the wild-type and Δ*fur*::*cat* strains were determined by the ΔΔ*C_T_* method, using the 16S rRNA *rrsH* gene as an internal control (71).

cDNA and primers prepared for qPCR (Table 2) were also used to determine the operon structure of the *mct* gene cluster. Ten microliters of cDNA was used as the template in a 50-μL *Taq* ThermoPol reaction mixture. Touchdown PCR was performed by annealing for two cycles at 64.5°C, two cycles at 59.5°C, and 15 cycles at 54.5°C. Five microliters of this reaction mixture was then used as the template in a second 50-μl PCR mixture with identical primers. The touchdown steps were eliminated in the second PCR, and annealing occurred at 54.5°C for 30 cycles. All reactions were also performed on p0.1229_3 plasmid DNA as proof of successful amplification and on a cDNA control prepared without reverse transcriptase to verify that the cDNA input was not contaminated with genomic DNA.

### In-frame deletions.

Previous work demonstrated that a fragment of the p0.1229_3 plasmid encompassing nucleotides 2850 through 7950 was sufficient to amplify Stx when cloned into pBR322 (15). In-frame deletions of individual ORFs in this vector, pBR322::mcc1229, were generated with NEB’s Q5 site-directed mutagenesis kit. Primers facing outward from the chosen ORF were designed with the NEBaseChanger tool and used with Q5 polymerase to amplify a linear fragment from pBR322::mcc1229 (Tables 1 and 2). This product was treated with the KLD (kinase, ligase, DpnI) enzyme cocktail to digest template DNA and recircularize the plasmid according to the manufacturer’s instructions. Constructs were verified by PCR of DH5α transformant colonies using VF/VR primers (Tables 1 and 2). Mutations were then confirmed by Sanger sequencing, and plasmids were electroporated into C600 as described above to ensure that no wild-type copies remained. Complementation of the in-frame deletion mutants was accomplished by fusing the promoter region directly upstream of *mctA* to the desired ORF. This fragment was cloned into pACYC184, replacing the vector’s tetracycline resistance gene. Primers for Gibson assembly are given in Table 2. Clones were verified by restriction digestion and by PCR and Sanger sequencing using the pACYC184 VF/VR primers (Table 2).

### Inhibition assays.

Microcin production was evaluated by measuring the inhibition of a target strain in agar overlays (72). The microcin-producing strain was spot-inoculated onto MH agar and incubated at 37°C for approximately 24 h. Plates were inverted over filter paper discs impregnated with 300 μL chloroform for 30 min to kill producing cells. Cultures of the target strains were then suspended to 0.05 optical density at 600 nm (OD_600_) units per mL in soft (0.7%) nutrient agar, poured on top of the plates, and allowed to solidify. After incubation overnight at 37°C, inhibition was noted by the presence of halos surrounding a microcin-producing colony. Zones of inhibition (ZOIs) were quantified by subtracting the diameter of the producing colony from the diameter of the clear zone surrounding it. Spontaneous mutants growing within the zones of inhibition were restreaked to purify and retested in agar overlays to confirm microcin resistance. Known microcin and colicin producers and their corresponding indicator strains were obtained from the NCTC reference set, kindly provided by Robert F. Roberts (44).

For inhibition assays using supernatants, plates were inoculated with the test strain in soft agar as described above. Fecal samples from mice colonized with 0.1229 or its derivatives were collected 1 day after inoculation with PA2, suspended in 100 to 200 μL LB, and centrifuged. Ten microliters of the supernatant was spotted on top of the test strain and allowed to dry before incubation overnight at 37°C.

### Whole-genome sequencing and bioinformatics.

Genomic DNA was extracted from cultures grown overnight using the DNeasy blood and tissue kit (Qiagen). Libraries were prepared using the Nextera XT kit (Illumina, San Diego, CA) and sequenced on the MiSeq platform, generating 2- by 250-bp reads. Reads were assembled in the Galaxy workspace with the SPAdes tool (73), and single nucleotide polymorphisms were identified using Snippy (74), in comparison to the reference genome assembly GCA_000335355.2. Putative Fur binding sites and promoter motifs were identified by analysis of the p0.1229_3 sequence with RSAT (75) and BPROM (76), respectively.

### Supernatant experiments.

The supernatants of E. coli 0.1229 and its derivatives were harvested after 16 h of shaking at 37°C and passed through 0.2-μm cellulose acetate filters (VWR Life Sciences, Radnor, PA). Assays to quantify Stx amplification were performed as previously described (15). Briefly, the test strain of E. coli was suspended in 1 mL of the spent supernatant to an OD_600_ of 0.05 and inoculated on top of solid LB agar in a 6-well plate (BD Biosciences Inc., Franklin Lakes, NJ).

For Stx assays, the test strains were E. coli O157:H7 isolates. PA2 (77) was used routinely as it demonstrated the greatest Stx amplification in previous experiments (13). EDL933 (46) was used in the event that a PA2 mutant could not be obtained. Strains were diluted to an OD_600_ of 0.05 in either broth or the filtered supernatant and inoculated on top of solid LB agar in 6-well plates. Cultures were then incubated statically at 37°C for 8 h. Aliquots of each culture were removed to measure the OD_620_, and the remaining volume was treated with 6 mg/mL polymyxin B for 5 min at 37°C to release intracellular Stx. Samples were then centrifuged for 5 min to pellet cell debris, and supernatants were collected and stored at −80°C until use in a receptor-based enzyme-linked immunosorbent assay (R-ELISA).

### R-ELISA.

Shiga toxin was detected in a receptor-based ELISA as previously described (14). A microtiter plate was first coated with 25 μg/mL ceramide trihexosides (Matreya Biosciences, Pleasant Gap, PA) in methanol. The methanol was evaporated, and the plate was subsequently blocked overnight with 4% bovine serum albumin in phosphate-buffered saline (PBS) containing 0.05% Tween 20 (PBST). Supernatant samples were diluted in PBS as necessary and added to the wells for 1 h, with gentle shaking at room temperature. Monoclonal anti-Stx2 antibody was purchased from Santa Cruz Biotech (Santa Cruz, CA) and added to the wells at 1 μg/mL for 1 h. Anti-mouse secondary antibody conjugated to horseradish peroxidase was purchased from MilliporeSigma (Burlington, MA) and also added at 1 μg/mL for 1 h. Between each of the preceding steps, the plate was washed five times with PBST. One-step Ultra TMB (3,3′,5,5′-tetramethylbenzidine; Thermo Fisher) was then used for detection. The plate was incubated for approximately 5 min before the reaction was stopped with the addition of 2 M H_2_SO_4_, and the *A*_450_ was measured (Multiskan FC; Thermo Fisher). A standard curve was established using serial dilutions of the lysate from PA11, a high-Stx2a producer (77). The concentration of Stx2a in E. coli O157:H7 samples was determined by comparison to this curve and is reported in micrograms per milliliter, normalized to the OD_620_ of each E. coli O157:H7 culture.

### Animal experiments.

Male and female Swiss Webster mice aged 3 to 5 weeks were raised in the University of Michigan germfree colony. They were housed in soft-sided bubble isolators or sterile Isocages and fed autoclaved water and laboratory chow *ad libitum*. Throughout the experiment, the mice received sterile food, water, and bedding to maintain germfree conditions, except for the infecting E. coli strains. All animal experiments were conducted with the approval of the University of Michigan Animal Care and Use Committee.

Mice were infected orally with ∼10^6^ CFU of each E. coli inoculum. In coinfection experiments, 0.1229 and its derivatives were inoculated first, followed by PA2 1 week later. Mice were weighed prior to each inoculation and just prior to euthanasia. They were evaluated daily for evidence of illness (dehydration, ruffled coat, or reluctance to move) and were euthanized 1 or 7 days after PA2 infection or when they became moribund. Prior to euthanasia, evidence of illness was recorded, and at necropsy, samples were collected for bacterial culture, Stx2 ELISAs, and histological examination.

For bacterial cultures, samples of the cecal contents were weighed, serially diluted in sterile LB, and cultured on sorbitol-MacConkey (SMaC) agar. PA2 is non-sorbitol fermenting and appears as white colonies on SMaC plates. Cultures from cocolonized mice were quantified based on the number of pink or white colonies. For the quantification of Stx2, the cecal contents were stored at −20°C until evaluation with a Premier EHEC ELISA kit (Meridian Biosciences Inc., Cincinnati, OH). The concentration of Stx2a was determined by comparison to the PA11 standard curve discussed above (77).
