# Supplementary material for: Mcc1229, an Stx2a-Amplifying Microcin, Is Produced In Vivo and Requires CirA for Activity
Source: Infect Immun. 2022 Feb 17;90(2):e00587-21. doi: 10.1128/iai.00587-21 (PMC8853679; doi:10.1128/iai.00587-21)
Supplement: Supplemental file 1 — Supplemental material. Download iai.00587-21-s0001.pdf, PDF file, 0.8 MB [file iai.00587-21-s0001.pdf]

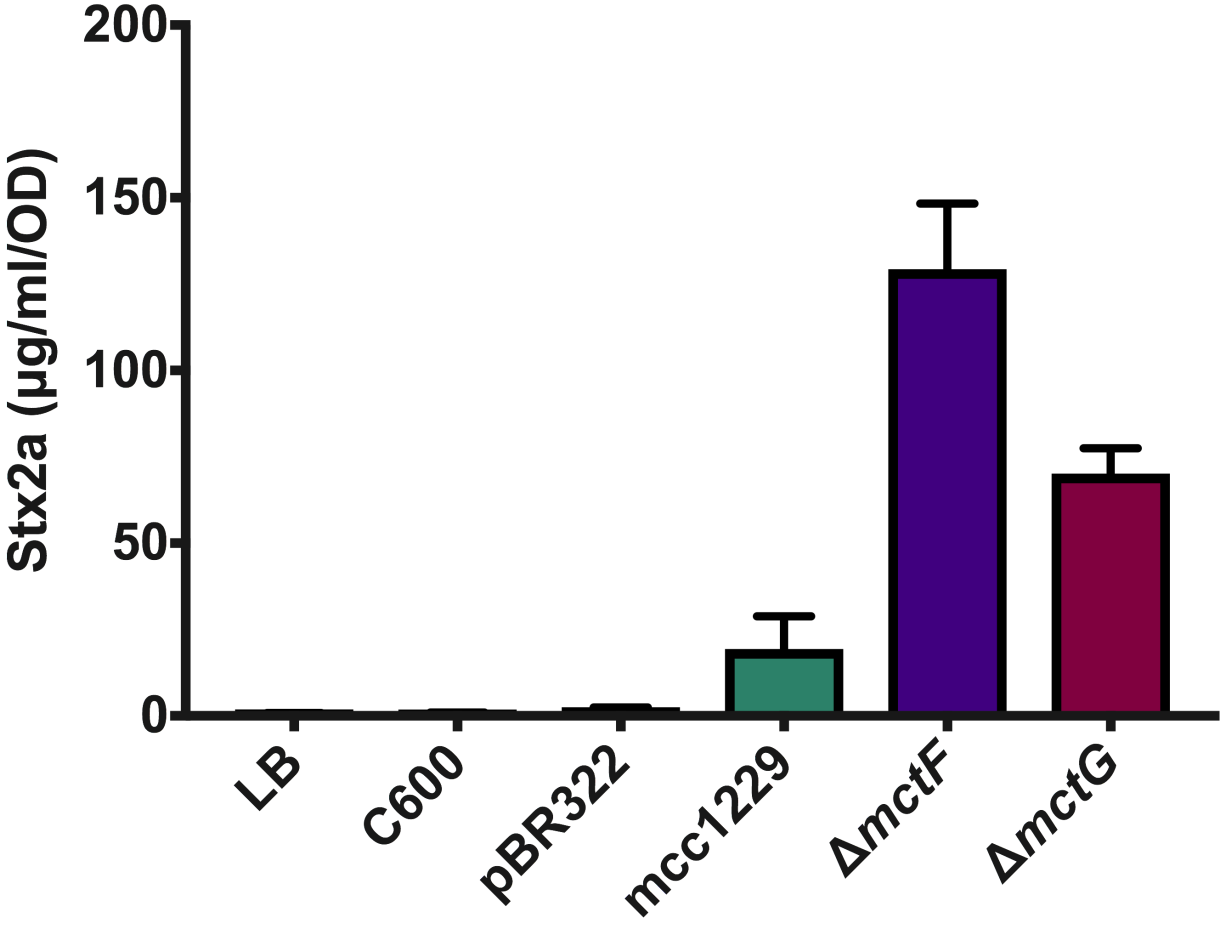

Fig S1. Deletions of *mctF* and *mctG* enhanced the Stx2a-amplifying activity of Mcc1229. The mcc1229 region of p0.1229_3 was cloned into pBR322 and was sufficient to amplify Stx2a. Supernatants from the C600 strain alone or from the empty vector pBR322 did not amplify Stx2a. Stx2a expression of PA2 exposed to filtered culture supernatants was determined by ELISA as previously described. LB represents the broth control, in which PA2 was grown in fresh medium rather than in filtered culture supernatant.

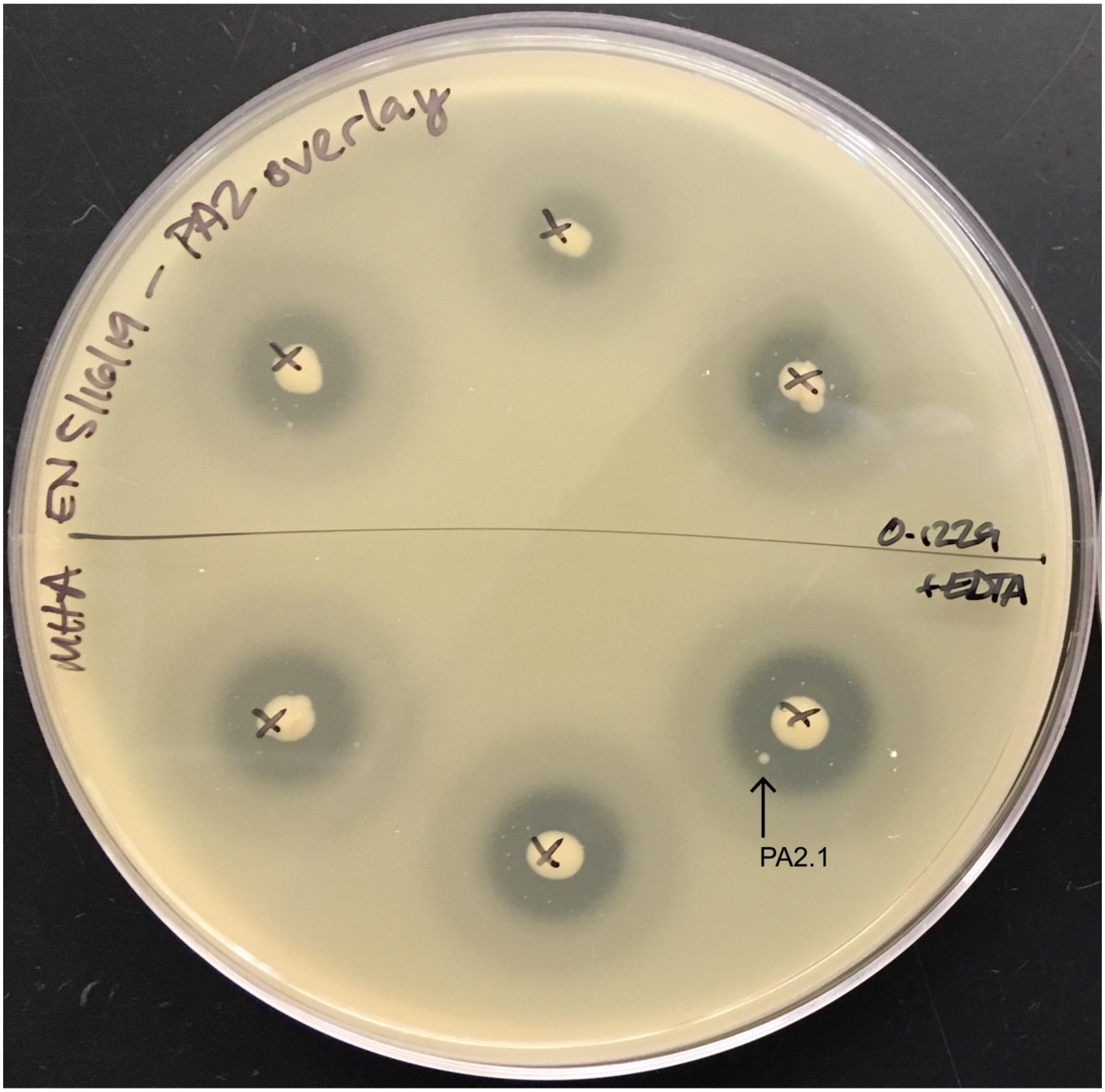

Fig S2. Spontaneous Mcc1229-resistant mutants grow within zones of inhibition (ZOI). Colonies of *E. coli* 0.1229ΔB17 were spotted on agar plates and allowed to grow for 24 h. The plates were then inverted over chloroform to kill Mcc1229-producing cells. *E. coli* PA2 was suspended in soft nutrient agar and poured atop the plate. Growth of PA2 was inhibited in zones immediately surrounding the microcin producers, but occasional spontaneous mutants (*e.g.* PA2.1) were recovered. Microcin-resistant colonies were restreaked to purify and were sequenced to determine the locus of resistance.
